# Non-specific aorto-arteriitis involving abdominal aorta branches in an adolescent girl (clinical case)

**DOI:** 10.1186/1546-0096-12-S1-P157

**Published:** 2014-09-17

**Authors:** Galina Glazyrina

**Affiliations:** 1 Hospital Pediatrics, South Ural State Medical University, chelyabinsk, Russian Federation

## Introduction

In children, the Takajasu arteriitis is one of the most frequent causes of renovascular hypertension.

## Objectives

To present a clinical case on non-specific aorto-arteriitis involving abdominal aorta branches in an adolescent girl.

## Methods

Patient Dina Zh., female, 14 years old.

The patient was admitted to the Department of cardiology and rheumatology of Chelyabinsk regional paediatric hospital on January 15, 2014. Fatigue, dyspnoe, headache, skin rash, BP increase up to 160/100 mm hg were reported. Severe disease was reported upon hospitalization; justification: Polyserositis: bilateral focal pleuropneumonia, class 2 respiratory failure, pancarditis (endomyopericarditis), class 2 heart failure, peritoneal exudation. Treatment resulted in stabilization; heart failure and respiratory failure symptoms were managed.

Treatment – arterial hypertension (130/80 – 160/110 mm hg) persisted. Pink – to purple livedo- like rash appeared on the lower extremities and abdominal wall. Proteinuria (daily loss up to 310 mg) was reported; diuresis level - 1350 ml/day. At hospitalization Day 3 hypertension attack was reported; it included BP increase up to 190/110 mm/hg, severe headache, spoor progressing to loss of consciousness, and seizures. CT of abdominal cavity with aortography: manifested narrowing of celiac trunk proximal regions, narrowing of superior mesenteric artery isthmus, narrowing of dextral renal artery. Blood circulation was not detected in proximal regions of the sinister renal artery. Inferior mesenteric artery: no significant changes reported.

Diagnosis: non-specific aorto-arteriitis (disease of Takajasu), stenosis type 2 involving celiac trunk, superior mesenteric artery, and renal arteries; secondary arterial hypertension, symptomatic epileptic seizures.

## Results

Treatment: pulse treatment with cyclophosphane (15 mg/kg dose), prednisolone (60 mg/day, i/m injections), anti-hypertension treatment, anti – aggregation treatment. Due to presence of abdominal aorta branches, critical stenosis the patient was transferred to the Federal centre of cardiovascular surgery of Chelyabinsk. Operation: stunting of the affected arteries; sinister renal artery angioplastics. No complications were reported during the post-operation period. Improvements were reported, including recovery of consciousness, and BP normalization; the patient resumed oral feeding, and manifestations of heart failure were managed. The patient’s condition has improved; the girl was active. Normal blood pressure and no complaints were reported. The patient demonstrated normal appetite; weight gain was reported. US imaging: recovery of renal circulation was reported. Treatment was continued: methylprednisolone (16 mg/day), methotrexate (12.5 mg/day), folic acid (1 mg/day), brilinta (180 mg/day), nifedipine (20 mg/day). The patient was discharged on March 14, 2013; satisfactory health status was reported.

## Conclusion

This case was associated with affection of abdominal aorta branches (renal arteries, celiac trunk, and superior mesenteric artery); thoracic and abdominal aortas were not involved. Latent disease progressed to manifestation phase after the development of arterial stenosis; development of the hypertension attack required emergency surgical intervention (stunting and angioplastics). Disease outcome was good.

## Disclosure of interest

None declared.

